# Binding mechanism and SERS spectra of 5-fluorouracil on gold clusters

**DOI:** 10.3389/fchem.2022.1050423

**Published:** 2022-12-05

**Authors:** Nguyen Thanh Si, Pham Vu Nhat, Minh Tho Nguyen

**Affiliations:** ^1^ Department of Chemistry, Can Tho University, Can Tho City, Vietnam; ^2^ Laboratory for Chemical Computation and Modeling, Institute for Computational Science and Artificial Intelligence, Van Lang University, Ho Chi Minh City, Vietnam; ^3^ Faculty of Applied Technology, Van Lang University, Ho Chi Minh City, Vietnam

**Keywords:** 5-fluorouracil, 5FU, SERS spectra, gold clusters, binding mechanism, binding energy, DFT

## Abstract

The adsorption behaviour of the 5-fluorouracil (5FU) on small gold clusters Au_*N*_ with *N* = 6, 8, 20 was evaluated by means of density functional theory using the PBE-D3 functional in combination with a mixed basis set, i.e. cc-pVDZ-PP for gold atoms and cc-pVTZ for non-metal elements. The binding energies between 5FU and gold clusters were determined in the range of 16–24 and 11–19 kcal/mol in gas-phase and aqueous media, respectively. The corresponding Gibbs energies were found to be around -7 to -10 kcal/mol in vacum and sigificantly reduced to -1 to -6 kcal/mol in water solution, indicating that both the association and dissociation processes are likely spontaneous. An analysis on the charge density difference tends to confirm the existence of a charge transfer from the 5FU molecule to Au atoms. Analysis of the surface-enhanced Raman scattering (SERS) spectra of 5FU adsorbed on the Au surfaces shows that the stretching vibrations of N−H and C=O bonds play a major role in the SERS phenomenon. A mechanism for the drug releasing from the gold surfaces is also proposed. The process is triggered by either the low pH in cancerous tumors or the presence of cysteine residues in protein matrices.

## Introduction

**FIGURE 1:**
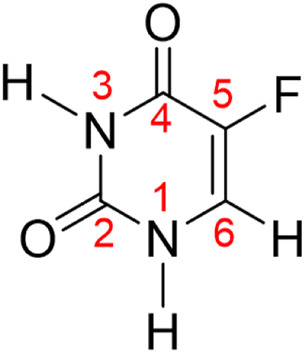
Chemical structure and atomic numbering of the drug molecule 5-fluorouracil (5FU).

The 5-fluorouracil molecule, denoted as 5FU in Figure 1, which is commercialized under the trade name Adrucil, is one of the most widely used drugs in the cytotoxic chemotherapy to treat different cancers such as those of the colon, stomach, pancreas, breast, and esophagus (Vodenkova et al., 2020). The practical use of this drug has been well documented in both *in vivo* and *in vitro* studies (Al-Jumayli et al., 2022; Khodarahmi et al., 2022), increasing the importance of the compound in the field of medicinal chemistry (Li et al., 2021). In view of its widespread use, it is necessary to focus on its increasing biocompatibility and reduction of its undesirable therapeutic effects. Some common side effects including loss of appetite, low blood cell count, hair loss, inflammation of the mouth and skin, *etc.* Have been found (de Oliveira et al., 2021; Solhjoo et al., 2021). When topically used, it can cause skin irritation, especially during pregnancy (Oliveira et al., 2021). Therefore, it is extremely important to use the highly biocompatible, hydrophilic carriers to reduce such inherently damaged issues (Frydenberg et al., 2020; Wang et al., 2021). In addition to an improvement of the therapeutic efficacy of 5FU and a reduction of adverse events, it is also crucial to quickly detect its presence in a biological environment by a simple detection method (Yuksel et al., 2022).

Drug carriers play an important role in reaching the target area in the body as well as improving the therapeutic effect by adjustment of the optimal dose (Román et al., 2018; Zahed et al., 2018). While most polar drugs do not access cell membranes, nanomaterials can help solving this problem because of their high surface-to-volume ratio and low density. Furthermore, the small size, high stability and easily suitable surface modification properties of these nanomaterials also encourage their use as drug carriers (Al-Musawi et al., 2019; Horo et al., 2019).

For instance, the ability of the boron-fullerene B40 and its exohedral metallic derivatives (Au@B40 and Au4@B40) to carry 5FU was recently evaluated by DFT computations (Afarideh et al., 2018). Up to four Au atoms can be attached on the outer surface of the B40 cage at once to obtain the unique capability for delivering this molecule. Each Au atom has the potential to interact with one 5FU molecule when using only one B40 molecule. Due to the low binding energies and charge transfer, the 5FU drug can easily be detached from B40, Au@B40, and Au4@B40 at room temperature. The Ni-doped (8,0) boron nitride nanotube (Ni-BNNT) was employed as a 5FU adsorbent and sensor because it improved adsorption ability (up to −17 kcal/mol) against 5FU (Sanad et al., 2019). Additionally, a large charge transfer occurs between 5FU and Ni-BNNT (Aal, 2022). Due to their advantageous characteristics, the pure and encapsulated B36N36 nanoclusters have been recommended as potential new delivery systems and detectors for 5FU drugs. An important stage is the nature of the interactions between drugs and various nano-based materials that have been the subject of numerous recent reports (Chen et al., 2022; Nguyen et al., 2022; Shakerzadeh et al., 2022; Yuksel et al., 2022).

Of the currently known nanomaterials such as carbon nanotubes (Minhas et al., 2021), boron nitride nanotubes (Kaur et al., 2019; Zarghami Dehaghani et al., 2021), chitin-chitosan (Sargazi-Avval et al., 2021), silica (Mirhaji et al., 2018), ninosome, the gold-based nanoparticles (Manzano and Vallet-Regí, 2020; Onishi et al., 2020; Siddique and Chow, 2020) are becoming attractive owing to their high biocompatibility, facile synthesis with many different sizes and shapes (Du et al., 2018; Khoshnevisan et al., 2018). These unique properties and applications in medicine have been discussed thoroughly in several recent reports (Elahi et al., 2018; Freitas de Freitas et al., 2018; Lee et al., 2020). In fact, gold nanoparticles have been approved as appropriate materials for many essential applications in biomedicine, biosensors and imaging (Bai et al., 2020; Bansal et al., 2020; Zhang et al., 2020). In this context, it is important to elucidate the interaction mechanism, as well as the binding nature, of the drug and gold nanoparticles (Sotnikov et al., 2019; Taghizadeh et al., 2019; Nhat et al., 2020a; Chen et al., 2021; Hang et al., 2021). It has been found that the interactions are mainly stabilized by a charge transfer from the electron-rich atoms of ligands to Au metals (Nhat et al., 2020b; Si et al., 2021). In some other cases, the gold atoms can act as a proton acceptor giving rise to nonconventional hydrogen bonds (Pakiari and Jamshidi, 2007; Nhat et al., 2020a). Nevertheless the interaction mechanism of gold nanoparticles is not well understood yet. From a methodological viewpoint, a question of interest is as to whether the particle surface could be modelled by some small-size clusters.

A majority of 80–85% of the administered 5FU drug is quickly converted to dihydroflourouracil during 5FU metabolism (Kryachko and Remacle, 2005). Furthermore, a tiny fraction (1–3%) of the drug given is transformed into fluorodeoxyuridine monophosphate and fluorouridine triphosphate, thereby inhibiting DNA synthesis and RNA processing. Urine excretes the remaining 12–19% of the unmetabolized 5FU form (Si et al., 2021). The concentration of 5FU in untreated wastewater was evaluated to be close to 18 ng/L (Tendwa et al., 2021) The ambient 5FU cytostatic drug concentration amounts to ∼0.21 ng/L (Mazzuca et al., 2016). Numerous analytical methods (Rowney et al., 2009) have been used to detect 5-fluorouracil in blood sample, such as polarography (Franquet-Griell et al., 2015), amperometric (Dos Santos et al., 2022), photoluminescence (Ibrahim et al., 2018), high-performance liquid chromatography (Hanif et al., 2018; Liu et al., 2019), and gas chromatography-mass spectrometry (Gui et al., 2013). These techniques are effective, but they are costly due to the large testing costs, have limited throughput and take a long detection time because the sample must undergo complicated treatment procedures before analysis. Therefore, it is now important to devise a quick, economic and convenient approach for the 5FU detection.

The surface-enhanced Raman scattering (SERS) phenomenon occurs when a Raman active molecule is adsorbed on the surface of a nanoparticle, considerably enhancing its Raman intensities (Schlücker, 2014). The electromagnetic (EM) and charge transfer (CT) mechanisms emerge now as the two main explanations for the enhancing mechanism of SERS. According to the EM approach, the enhancement is mostly due to interactions between molecules and the massive surface resonance plasmons that an incoming laser causes to form on the substrate’s surface (Mullot et al., 2009). Following the CT approach, the increase also involves a significant charge transfer as during the associated electronic resonance transition between the molecules and the substrate (Schlücker, 2014). For the detection of a single molecule, the SERS technique appears to be a highly sensitive analytical technique because of its high enhancement efficiency (Weiss and Haran, 2001).

In this context, we set out to carry out in present study quantum chemical calculations with the aim to evaluate the binding mechanism between gold nanoparticles and the 5FU molecule by using the gold Au_*N*_ clusters with *N* = 6, 8, 20 as models for the gold surfaces. In addition, the surface enhanced Raman scattering (SERS) mechanism and the applicability of gold nanoparticles for detection of the 5FU molecule are also elucidated.

## Computational methods

All electronic structure calculations in this study are performed using density functional theory (DFT) with the PBE-D3 functional (Perdew et al., 1996; Grimme et al., 2010) including the dispersion correction (D3) in conjunction with the effective core potential (ECP) cc-pVDZ-PP (Peterson, 2003) for gold atoms and the cc-pVTZ basis set for other atoms using the Gaussian 16 package (Frisch et al., 2019). In the cc-pVDZ-PP basis set, the inner electrons along with the nucleus are considered as an inert core, and those on 5 *s*, 5*p*, 5 *d*ays, and 6Is orbitals of the Au atom are taken as valence electrons. The interaction between valence electrons and inert core is included in the pseudopotential which also takes care of the relativistic effects of the heavy gold atoms. This allows us to reduce greatly the number of electrons to be treated, and thereby computing times, but still describe properly the interactions between gold atoms and biomolecules (Nhat et al., 2020a; Nhat et al., 2020b). In addition, the Integral Equation Formalism-Polarizable Continuum Model (IEF-PCM) (Tomasi et al., 2005) is employed to investigate the effects of aqueous solvents. The density of states (DOS) are simulated by using the GaussSum program (O'boyle et al., 2008). The charge density difference (CDD) maps and energy decomposition analyses are obtained by using the Multiwfn 3.8 software (Lu and Chen, 2012).

The binding energies (E_b_) is simply defined as the energy difference between those of the Au_*N*_-5FU complexes with respect to the free 5FU drug molecule and the Au_*N*_ (*N* = 6, 8, 20) species:

$$E_{b}=E_{{Au}_{N}-5FU}-\left(E_{{Au}_{N}}+E_{5FU}\right)$$

where $E_{{Au}_{N}-5FU}$ is the total energy of the $E_{{Au}_{N}-5FU}$ complexes, $E_{{Au}_{N}}$ and $E_{5FU}$ the total energies of Au_*N*_ (*N* = 6, 8, 20) clusters and 5FU drug, respectively. Thus, a negative value of $E_{b}$ corresponds to an exothermic adsorption.

The Gibbs free energy of the interaction are calculated as follow:

$${∆G}^{ο}={\left(E+G_{corr}\right)}_{{Au}_{N}-5FU}-\left[{\left(E+G_{corr}\right)}_{{Au}_{N}}+{\left(E+G_{corr}\right)}_{5FU}\right]$$

where $\left(E+G_{corr}\right)$ is the sum of electronic and thermal free energies.

The energies of frontier states such as HOMO and LUMO, and the derivative HOMO–LUMO band gap (Eg) are also computed to examine the electronic responses during adsorptions. The Eg is a useful factor for determining the kinetic reactivity of materials (Hadipour et al., 2015), and its change upon the adsorption process can be used to evaluate the sensitivity of absorption.

## Results and discussion

### Structures of 5FU drug and gold clusters

**FIGURE 2:**
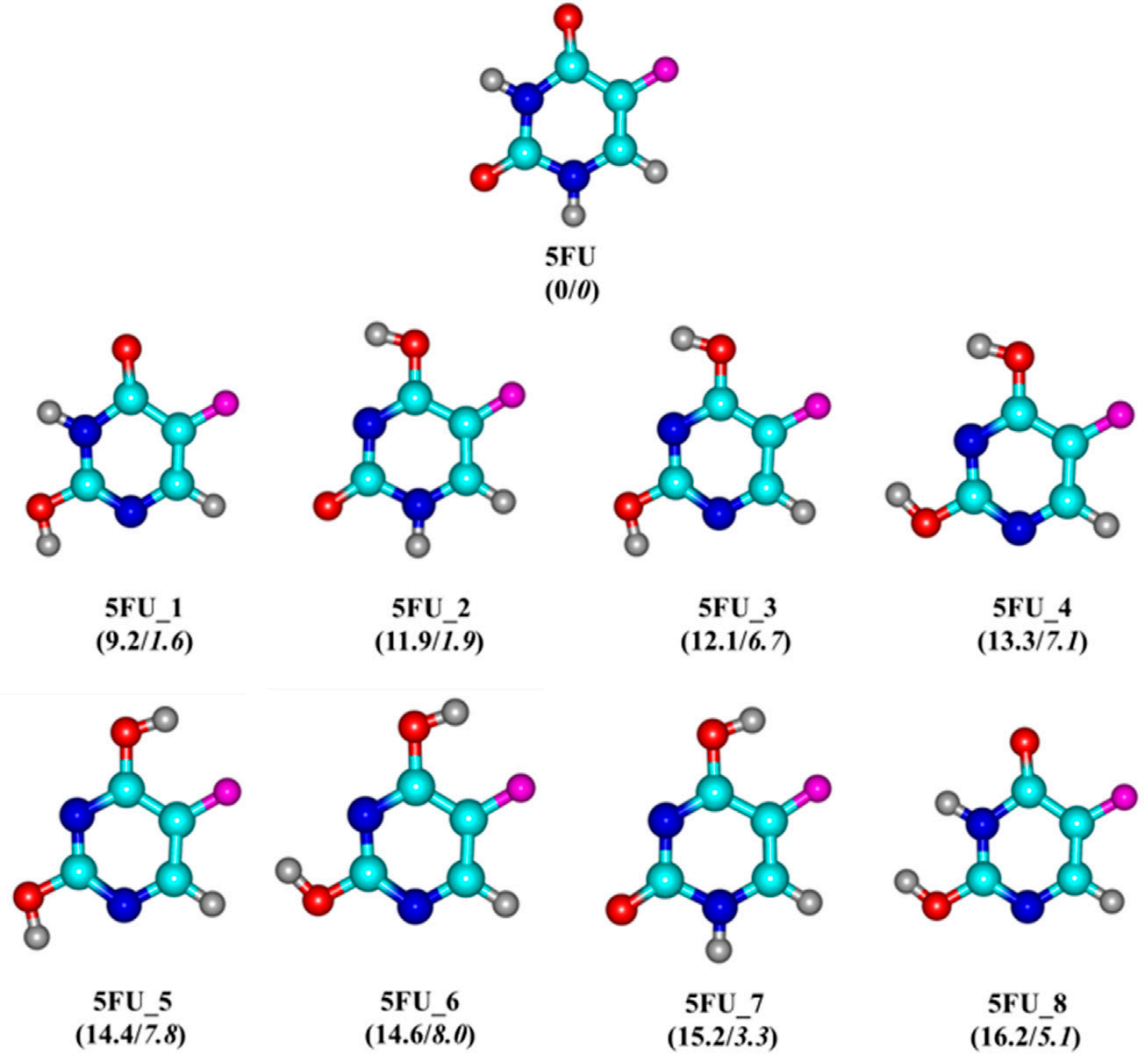
Optimized structures of 5FU and its tautomers. Values (kcal/mol) given in parentheses represent relative energies of 5FU tautomers at the PBE-D3/cc-pVTZ + ZPE level in vacuum/water solution with respect to 5FU.

Several theoretical and experimental studies (Scanlan and Hillier, 1984; Hadipour et al., 2015; Wielińska et al., 2019) have previously been conducted to determine the chemical structure of 5FU drug and at least eight different tautomers have been located (*cf.* Figure 2). Accordingly, the molecule exhibits a planar structure, containing a six-membered N-heterocylic ring (pyrimidine ring) with a fluorine substituent at the C5 position. This modification process is completely similar to that of DNA bases as a result of the hydrogen transfer within oxygen and nitrogen atoms (Pascu et al., 2005; Markova et al., 2010).

Generally, the proton transfer in the 5FU molecule results in eight possible tautomers (Figure 2), including one di-keto (5FU), four keto-enols (5FU_1, 5FU_2, 5FU_7 and 5FU_8), and three di-enols (5FU_3, 5FU_4 and 5FU_5). The two rotational conformations (5FU_7 and 5FU_8) are also found upon rotation of the O−H group around the C-O group (5FU_1, 5FU_2). This is similar to that of 5FU_4 and 5FU_5 isomers. At the PBE-D3/cc-pVTZ level, the 5FU structure is 9–16 kcal/mol more stable than its tautomeric forms (gas-phase values). Such an energy gap is reduced to 1–8 kcal/mol in the aqueous solution. These computed results are fully consistent with both theoretical and experimental results previously reported (Scanlan and Hillier, 1984; Markova et al., 2010). The predicted distance of the C−F bond is 1.341 Å, which is comparable to the experimental value of 1.348 Å (Rastogi et al., 2000). The bond lengths of C2 = O and C4 = O are computed to be 1.220 and 1.221 Å, respectively, being somewhat longer than the experimental values of 1.212 and 1.211 Å (Rastogi and Palafox, 2011).

**FIGURE 3:**
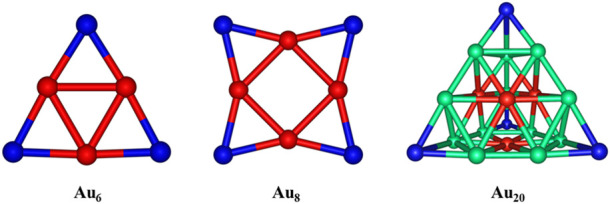
NBO charge distributions in Au_*N*_ (*N* = 6, 8, 20) clusters. Color range given in a.u. Are: blue: more positive than 0.06; red: more negative than -0.06.

As reported earlier, the Au_6_ and Au_8_ clusters have planar structures, while the Au_20_ cluster gas prefers a pyramidal shape in their ground states (Rastogi and Palafox, 2011). To determine the favored binding sites between the gold clusters and the 5FU molecule, an analysis of NBO charge distribution (Rastogi et al., 2000) is first caried out and presented in Figure 3. The corner Au atoms (blue color) are found to bear a more positive charge than others (red color). According to previous studies (Nhat et al., 2017; Nhat et al., 2020b), the 5FU molecule is expected to interact with gold clusters by anchoring its electron-rich sites on the positively charged atoms of gold surfaces. In addition, the binding is in part stabilized due to the formation of some nonconventional X−H···Au hydrogen bonds. To evaluate the dynamic stability of the gold cluste-5FU complexes we carry out Born-Oppenheimer molecular dynamics simulations for Au_6_-5FU and Au_8_-5FU systems at 300 K. Simulation results that are collected in Figure S3 (SI file), show the connectivity between atoms in these systems remains unchanged during the simulation time, indicating that they are dynamically stable.

### Absorption of 5FU drug on gold cluster surface: Structures and energies

**FIGURE 4:**
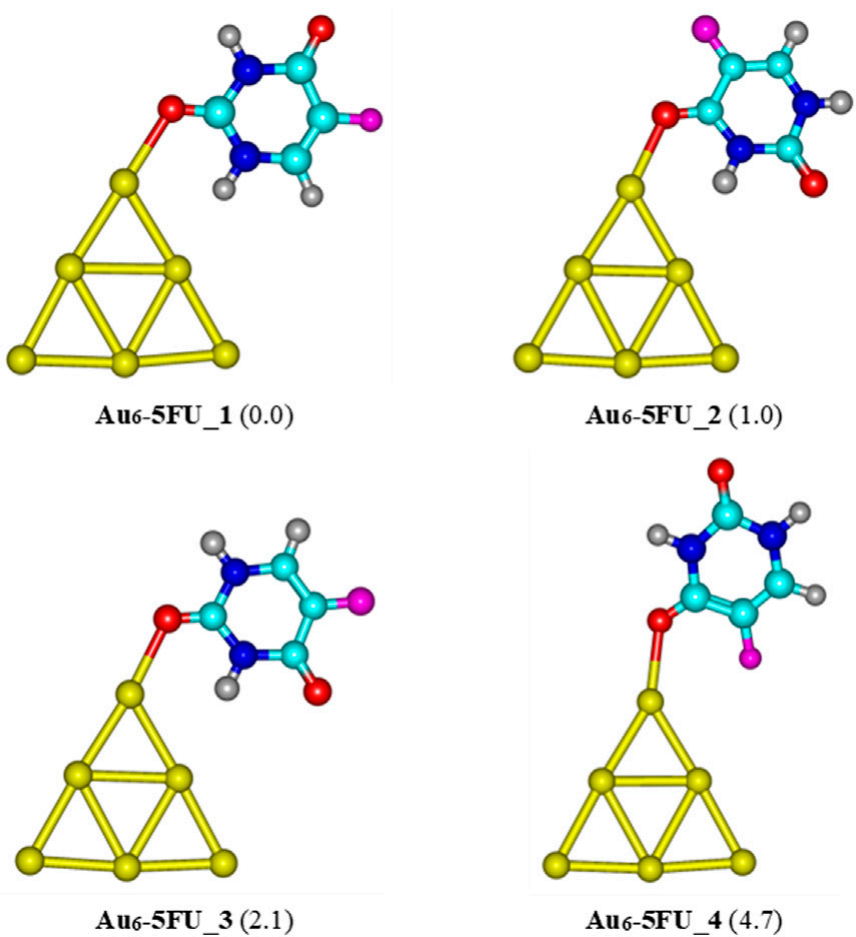
Low-lying structures of the Au_6_-5FU complex in gas-phase. Values given in parentheses are relative energies (kcal/mol) with respect to the most stable form Au_6_-5FU_1. Yellow, cyan, blue, white-gray, red, and magenta are used to represent the Au, C, N, H, O, and F atoms, respectively.

**FIGURE 5:**
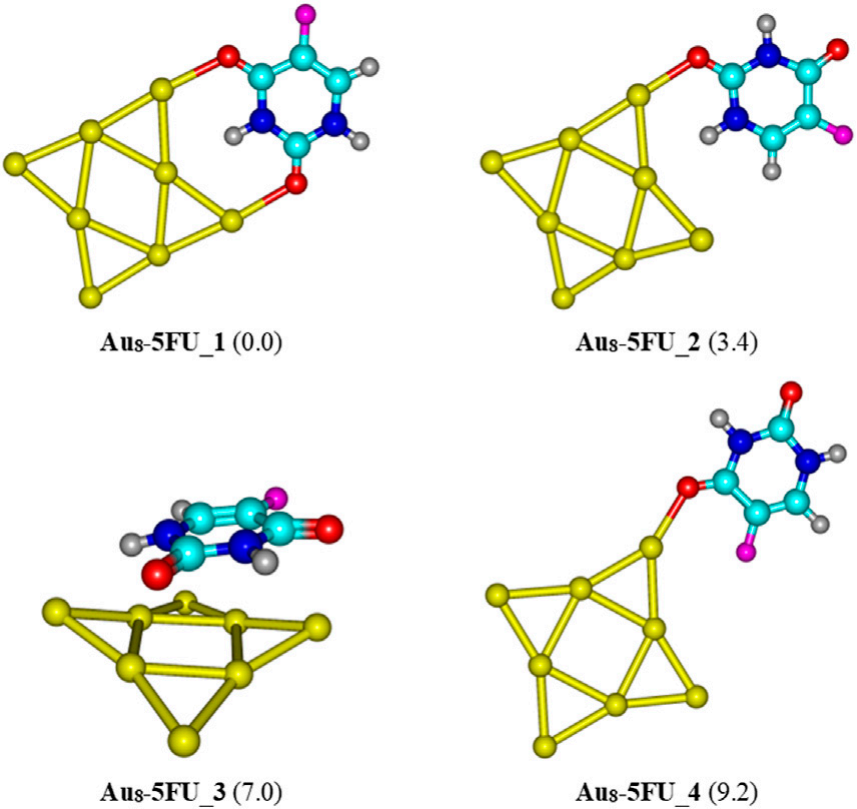
Low-lying structures of the Au_8_-5FU complex in gas phase. Values indicated in parentheses are the relative energies (kcal/mol) with respect to the most stable isomer Au_8_-5FU_1. Yellow, cyan, blue, white-gray, red, and magenta are used to represent the Au, C, N, H, O, and F atoms, respectively.

**FIGURE 6:**
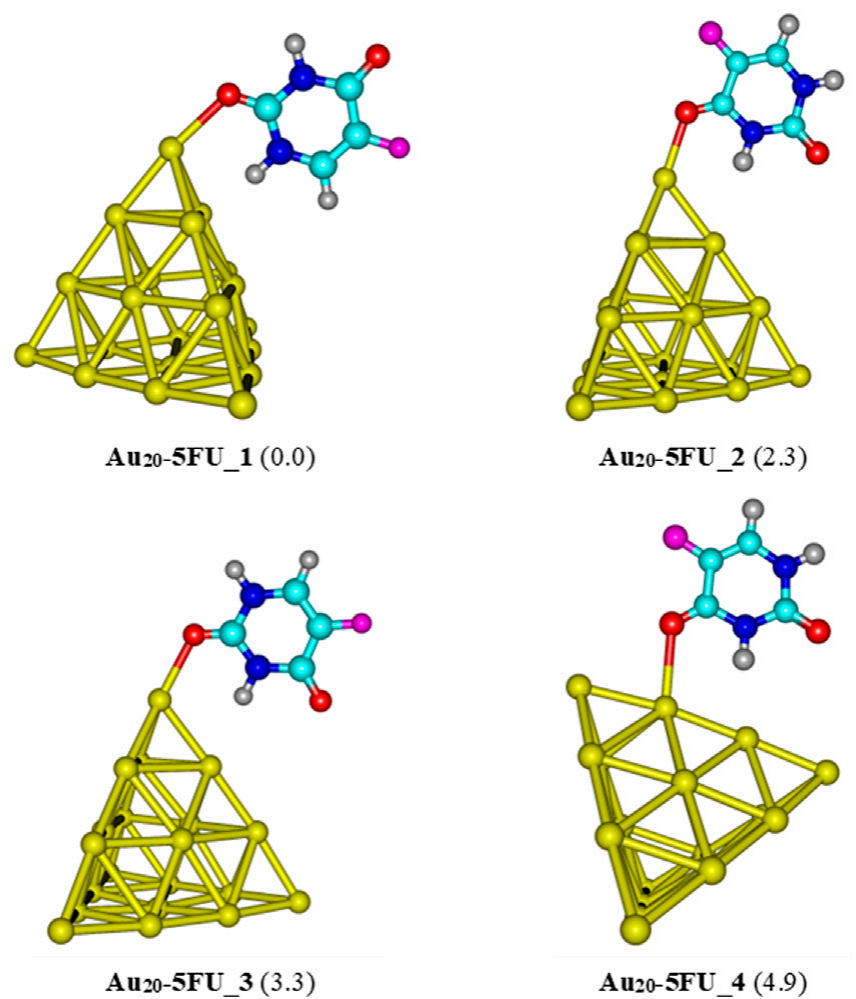
Low-lying structures of the Au_20_-5FU complex in the gas phase. Values indicated in parentheses are the relative energies (kcal/mol) with respect to the most stable structure Au_20_-5FU_1. Yellow, cyan, blue, white-gray, red, and magenta are used to represent the Au, C, N, H, O, and F atoms, respectively.

Four structures of Au_*N*_-5FU (*N* = 6, 8, 20) complexes obtained at the PBE-D3/cc-pVTZ/cc-pVDZ-PP level are displayed in Figure 4−6. Accordingly, the most stable structure of Au_6_-5FU complexes contains an Au−O bond constructed by anchoring the oxygen atom of the C2 = O group on the corner of Au_6_. This structure is almost planar with an Au−O bond length of 2.317 Å. The oxygen atom of the C4 = O group is the next preferred site for Au_6_ attack, resulting in the Au_6_-5FU_2 complex. This form is ∼1.0 kcal/mol less stable than Au_6_-5FU_1. The two remaining structures, namely Au_6_-5FU_3 and Au_6_-5FU_4, that can be considered as rotational isomers of Au_6_-5FU_1 and Au_6_-5FU_2, respectively, are lying around 2–5 kcal/mol above. A perpendicular anchor type between the Au_6_ cluster and the 5FU molecule is not found.

As in Au_6_-5FU, the oxygen atom of the C2 = O and C4 = O carbonyl groups is also the most preferred site for 5FU binding to Au_8_ (Figure 5). The lowest-energy Au_8_-5FU_1 has a binding energy of −24 kcal/mol, as compared to a corresponding value of −17 kcal/mol obtained for Au_6_-5FU_1 (Figure 4). This structure is stabilized mainly due to interaction of 2 C=O bonds with the Au_8_ cluster. The next stable complex Au_8_-5FU_2 is ∼3.4 kcal/mol higher in energy than Au_8_-5FU_1. Noticably, we find a new type of adsorption, i.e. Au_8_-5FU_3, in which the 5FU molecule is lying parallelly on the gold surface. However, such interaction induces a slight change in geometry of Au_8_ cluster. Similar to the Au_6_-5FU system, the C4 = O group is not a preferred site for binding to the gold atoms. Indeed, this binding pattern (Au_8_-5FU_4) has a relative energy of ∼9 kcal/mol higher than the most stable form.

For the interaction between the pyramidal Au_20_ and 5FU, four stable structures are identified and presented in Figure 6. The most stable form Au_20_-5FU_1 has a binding energy of around −17 kcal/mol. The next stable complex Au_20_-5FU_2 is formed by anchoring the O atom of the C4 = O group on a corner of Au_20_ is computed to be ∼2.3 kcal/mol less stable than Au_20_-5FU_1. The structure Au_20_-5FU_3 is also rather stable as located at ∼3 kcal/mol above Au_20_-5FU_1 and is basically considered as a rotational isomer of the latter. On the contrary, the resulting complexes that are formed by adsorption of 5FU on an Au atom at the edge of the Au_20_ cluster are much less stable, being ∼5 kcal/mol higher in energy.

Table 1 presents the calculated binding energy (E_b_) and Gibbs free energy (∆G) for the 5FU adsorption on gold clusters obtained at the PBE-D3/cc-pVTZ/cc-pVDZ-PP level. The adsorption processes are exothermic indicating all stabilizing interactions. However, due to entropic effects, i.e. entropy is decreased during the adsorption, the Gibbs free energies turn out to be less negative as compared to corresponding binding enthalpies. In particular, the higher reactivity toward the 5FU molecules of Au_8_ in comparison with Au_6_ and Au_20_ clusters correlates with its smaller HOMO–LUMO gap and lower stability (Nhat et al., 2020a).

**TABLE 1:** Relative energy (RE), binding energy (E_b_) and Gibbs free energy (ΔG) for the 5FU adsorption on gold clusters Au_*N*_ with *N* = 6, 8, 20 in their optimized complexes (PBE-D3/cc-pVTZ/cc-pVDZ-PP, kcal/mol).

Structure	Re		E_b_		ΔG	
	In gas phase	In water	In gas phase	In water	In gas phase	In water
Au6-5FU_1	0.0	0.8	−16.8	−12.6	−6.5	−3.0
Au6-5FU_2	1.0	0.0	−15.8	−13.4	−5.7	−2.1
Au6-5FU_3	2.1	0.6	−14.7	−12.8	−4.8	0.1
Au6-5FU_4	4.7	2.6	−12.1	−10.7	−4.0	0.3
Au8-5FU_1	0.0	0.0	−23.5	−19.1	−9.7	−6.3
Au8-5FU_2	3.4	0.0	−20.1	−15.3	−8.2	−3.6
Au8-5FU_3	7.0	2.9	−16.4	−16.2	−4.9	−2.0
Au8-5FU_4	9.2	6.1	−14.3	−13.0	−5.1	−5.2
Au20-5FU_1	0.0	1.8	−16.8	−11.3	−7.5	−1.5
Au20-5FU_2	2.3	0.7	−14.5	−12.4	−3.5	−1.9
Au20-5FU_3	3.3	0.0	−13.5	−13.1	−2.5	−2.8
Au20-5FU_4	4.9	2.4	−11.8	−10.7	−1.6	0.0

When including the influence of an aqueous solvent, both E_b_ and ΔG values become less negative than those computed in vacuum. The numerical data obtained for interactions of 5FU molecule and Au_*N*_ (*N* = 6, 8, 20) clusters in a water environment presented in Table 1 show that the Au_8_ cluster also has the highest affinity with the 5FU molecule.

In the most stable complexes Au_*N*_-5FU_1 (*N* = 6, 8, 20), the bond lengths of Au−O amount to 2.317, 2.359 (2.417), and 2.404 Å in gas phase and 2.329, 2.337 (2.370), and 2.397 Å in water, respectively. Thus it can be seen that the bidentate formation of Au−O bonds in Au_8_-5FU_1 correlates well with its largest bonding energy. Overall, the bond lengths are predicted to be slightly longer than the sum of the covalent radii of oxygen (0.73 Å) and gold (1.44 Å) atoms (Pakiari and Jamshidi, 2007).

### Electronic properties of frontier orbitals

For deeper insights into the interaction mechanisms, we now consider the energies of frontier orbitals, namely the HOMO and LUMO, in both isolated components and resulting products. The change of the HOMO−LUMO energy gap (ΔEg) of gold clusters during the 5FU adsoption is computed using the following equation:

$$\Delta E_{g}=\frac{E_{2}-E_{1}}{E_{1}}\times 100$$

where $E_{1}$ and $E_{2}$ are the band gaps in Au_N_ clusters and Au_*N*_-5FU_1 complexes, respectively.

In gas-phase, the band gap of Au_8_ is predicted to be 1.4 eV (Table 2), which is significantly smaller than the corresponding values of 2.1 and 1.8 eV for Au_6_ and Au_20_, respectively. The result is thus correlates well with the largest binding energy of 5FU to Au_8_ cluster as analyzed above. Energies of frontier orbitals in addition provide us with important information on the binding mechanism. During the adsorption, a charge transfer could be processed from the 5FU molecule to the gold atoms, or reversedly depending on the energy gap between frontier states of adsorbent and adsorbate.

**TABLE 2:** Energies (eV) of frontier orbitals (HOMO and LUMO), their band gap (Eg) and change of Eg upon 5FU adsorption on gold clusters.

Structure	In gas phase					In water					
	HOMO	LUMO	E_*F*_	E_g_	%∆E_g_	HOMO		LUMO	E_*F*_	E_g_	%∆E_g_
5FU	−6.12	−2.44	−4.28	3.67		−5.95		−2.28	−4.12	3.67	
Au6	−5.94	−3.85	−4.89	2.10		−5.36		−3.07	−4.22	2.29	
Au6-5FU_1	−5.68	−3.77	−4.72	1.91	−8.94	−5.24		−3.20	−4.22	2.04	−11.00
Au8	−5.83	−4.39	−5.11	1.44		−5.30		−3.68	−4.49	1.62	
Au8-5FU_1	−5.21	−3.67	−4.44	1.54	+7.22	−5.11		−3.49	−4.30	1.61	−0.44
Au20	−5.74	−3.94	−4.84	1.79		−5.07		−3.18	−4.13	1.89	
Au20-5FU_1	−5.63	−4.00	−4.82	1.64	−8.80	−4.99		−3.19	−4.09	1.79	−5.25

**FIGURE 7:**
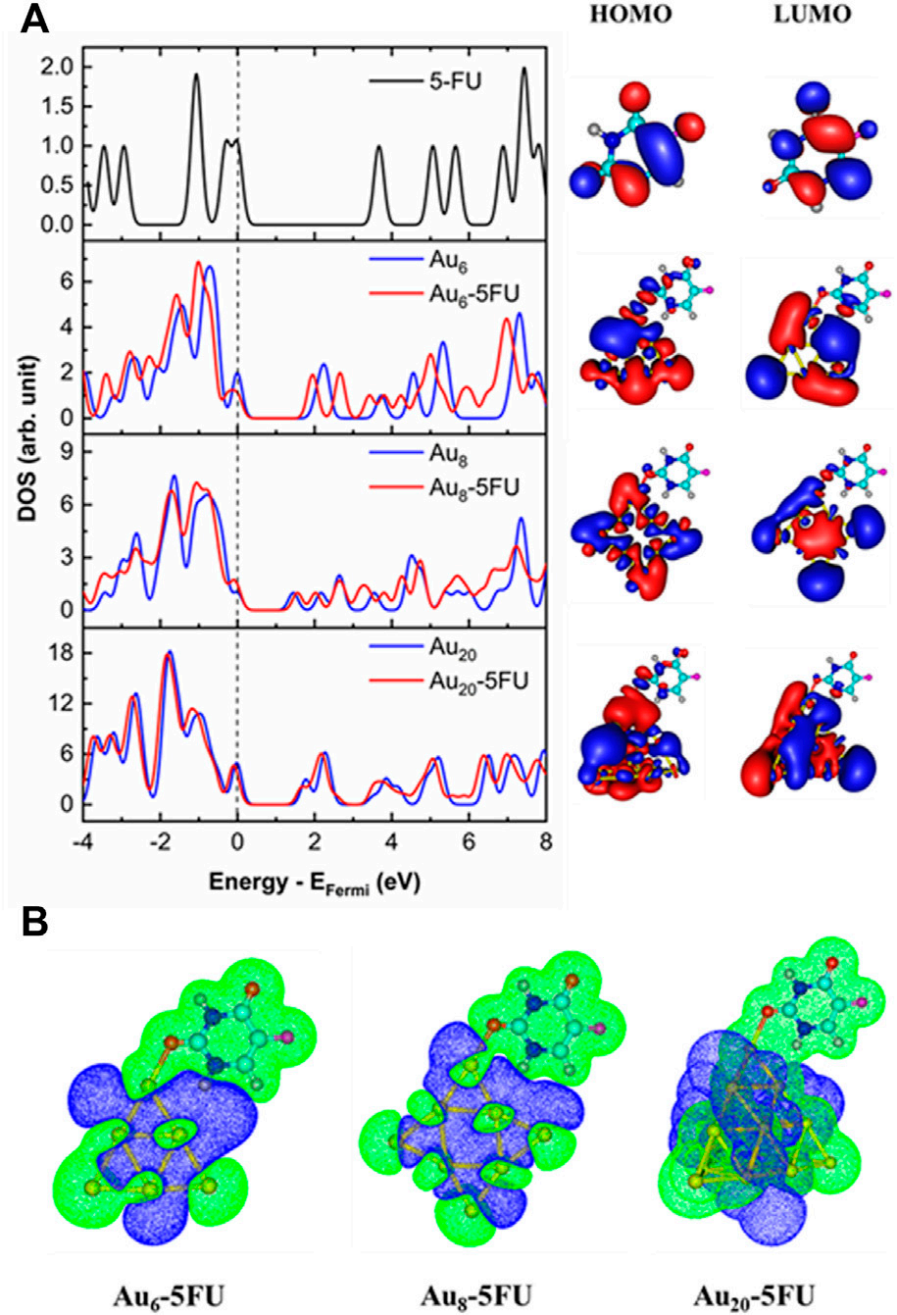
Density of states, HOMO and LUMO **(A)** and charge density difference maps **(B)** for the most stable complexes, Au_*N*_-5FU_1. The Fermi energy (E_Fermi_) level is corrected to the zero reference. Isovalue is equal to 0.01 au for MOs and to 0.005 au for CDDs.

In the gas phase, the energy difference between the HOMO of 5FU and LUMO of gold clusters amounts to about 1.7–2.3 eV, being smaller than that involving the HOMO of gold clusters and LUMO of 5FU, which ranges from 3.3 to 3.50 eV. This leads to a decrease in the band gaps of the gold clusters after adsorption with 5FU (Figure 7A). Thus, the charge densities are mainly transferred from the HOMO of the 5FU molecule to the LUMO of a gold cluster. Such a prediction is also elucidated by the charge density difference (CDD) map analysis (Figure 7B). The CDD is computed by the folowing equation:

$$∆\rho =\rho_{complex}-\rho_{{Au}_{N}}-\rho_{5FU}$$

where ρ is charge density and ∆ρ calculated by Multiwfn sofware. (Lu and Chen, 2012) Here, green and blue regions correspond to positive and negative regions, respectively, which also represent a decrease and an increase in electron density due to the adsorption.

A topological analysis for the most complexes Au_*N*_-5FU_1 (_*N*_ = 6, 8, 20) is performed and shown in Supplementary Table S1 and Supplementary Figure S1 (Supplementary File S1). The presence of bond critical points (BCPs) between Au, O and H atoms involved in intermolecular interactions in their complexes, as shown in Supplementary Figure S1, suggests that they occur. Two interactions between the gold and O, H atoms Au⋅⋅⋅O, Au⋅⋅⋅H are mainly responsible for these complex stabilization (Supplementary Figure S1). Such interactions have electron densities *ρ*_r_ that are quite low, 0.014–0.020 a.u. For Au⋅⋅⋅H and 0.049–0.053 a.u. For Au⋅⋅⋅O, indicating very weak interactions. As a result, for example Au_8_-5FU_1 complex, intermolecular interaction energy (E_int_) of these bonds around -11 kcal/mol (see in SI file). The Laplacian of electron density ( ∇ ^2^ *ρ*_r_) and total electron density energy (*H*_r_) are also important parameters for evaluating the type of non-covalent interactions (NCI). Supplementary Tables S1 shown the ∇ ^2^ *ρ*_r_ < 0 and *H*_r_ < 0, this confirmed to be partially covalent character. Finally the reduced density gradient (RDG) which is also an effcient method for analyzing the NCI, arre described in the SI file.

The energy decomposition analysis (EDA) is considered as an effective method for a quantitative interpretation of chemical bonding in terms of three main components (Morokuma, 1971; Ziegler and Rauk, 1977; Zhao et al., 2018). Accordingly, the instantaneous interaction energy ΔE_int_ between two fragments A and B in a molecule A–B is partitioned in three terms including 1) the quasi-classical electrostatic interaction ΔE_elstat_ between the fragments; 2) the repulsive exchange (Pauli) interaction ΔE_Pauli_ between electrons of the two fragments having the same spin, and 3) the orbital (covalent) interaction ΔE_orb_ which comes from the orbital relaxation and the orbital mixing between the fragments. The latter term can be decomposed into contributions from orbitals with different symmetry which make it possible to distinguish between σ, π, and *δ* bonding. Thus, ΔE_int_ is calculated according to the following expression (Liu, 2007):

$$∆E_{\mathrm{int}}=∆E_{elstat}+∆E_{Pauli}+∆E_{orb}=∆E_{steric}+∆E_{orb}$$

Our EDA analysis results are performed using Multiwfn software (Lu and Chen, 2012). For example, the interaction energy of Au_8_-5FU complex is -24 kcal/mol. The orbital interaction energy -41 kcal/mol significantly stabilizes the product; while the steric term (sum of electrostatic interaction energy, Pauli repulsion energy and change in exchange-correlation energy), destabilizes the adduct by 17 kcal/mol (see Supplementary Table S2 in SI file).

### SERS spectra of 5FU on gold surfaces

**FIGURE 8:**
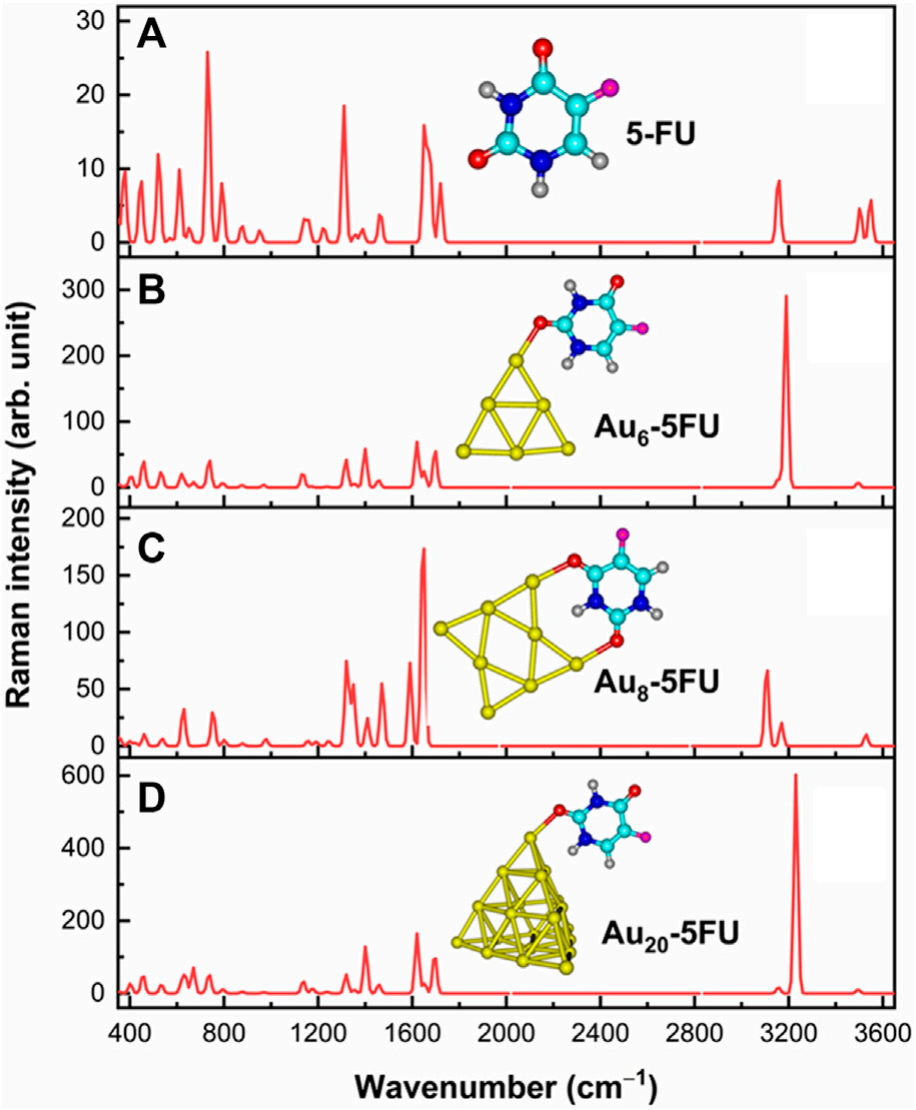
Raman signatures of 5FU **(A)** and its SERS spectra on Au_*N*_ (N = 6, 8, 20) surfaces **(B–D)** in aqueous solution.

Analyses of the vibrational signatures and the surface-enhanced Raman scattering (SERS) spectra are the effective tools for the rationalization of the drug binding mechanism on nanoparticles surfaces. In principle, Raman spectroscopy allows us to detect vibrational features of functional groups in organic compounds. However, when molecules are present at very low concentrations, this technique is naturally restricted due to the intrinsically weak Raman intensity. In this situation, the Raman intensities are often magnified by the use of the SERS techniques (Mohammadi et al., 2018). In order to interpret the SERS phenomenon, both chemical and electromagnetic enhancement mechanisms are frequently taken into accounts (Farquharson et al., 2008). A number of theoretical and experimental studies on the Raman and SERS spectra of 5FU molecule have been carried out (Farquharson et al., 2005; Cialla et al., 2014). In the present study, both Raman and SERS spectra of the 5FU molecule and its complexes in the range of 400–3600 cm^−1^ are simulated and illustrated in Figure 8, while details of some significant signals are provided in Table 3. The experimental data for free 5FU are also included in Table 3 for a comparison.

**TABLE 3:** Selected Raman frequencies (in cm^−1^) and intensities (in arbitrary unit) for 5FU and its complexes Au_6_-5FU_1 computed in aqueous solution.

Modes*	5FU			Au_6_-5FU_1		Au_8_-5FU_1		Au_20_-5FU_1	
	Expt	Position	Intensity	Position	Intensity	Position	Intensity	Position	Intensity
*ν*(N1−H)	3146	3548	6	3188	290	-	-	3230	602
*ν*(N3−H)	3022	3503	5	-	-	3106	56	-	-
*ν*(C2 = O)	1725	1719	8	1620	69	1645	164	1700	95
*ν*(C4 = O)	1672	1675	11	1697	54	1591	73	1646	160
*β*(N1−H)	1504	1464	8	1399	58	1472	55	1398	128
*β* (C6−H)	1258	1310	18	1318	42	1322	74	1373	60
*δ*(ring)	738	731	25	737	36	753	29	731	49

The Raman spectrum of 5FU is basically characterized by typical signals in the regions of 400–800, 1000–1800 and 3000–3600 cm^−1^. Several peaks located above 3000 cm^−1^ are due to the ν(C−H), ν(N1−H) and ν(N3−H) stretching modes. In particular, the calculated ν(C−H) stretching at 3156 cm^−1^ can be assigned to the experimental value of 3067 cm^−1^ (Farquharson et al., 2008). Both ν(N1−H) and ν(N3−H) modes are predicted to stretch at 3547 and 3503 cm^−1^. Because the H atom in the N3−H bond is involved in an intermolecular hydrogen bond, the wavenumber at 3503 cm^−1^ of ν(N3−H) is smaller than that of ν(N1−H), which is consistent with the experimental data of 3022 (N3−H bond) and 3146 cm^−1^ (N1−H bond) (Pavel et al., 2006). However, the calculated results of these bond stretchings are larger than the experimental counterpart (Rastogi et al., 2000).

Other important bands are related to the C=O stretching vibrations. As shown in Figure 8, these modes are observed at 1719 and 1675 cm^−1^, which are comparable to the experimental values of 1725 cm^−1^ and 1672 cm^−1^ (Rastogi et al., 2000). On the contrary, the peaks centered at 1652 and 1224 cm^−1^ that arise from the C=C and C−F stretching modes, agree well with the measured values at 1658 and 1224 cm^−1^, respectively (Rastogi et al., 2000). In particular, the highest peak observed at 731 cm^−1^ is due to the pyrimidine ring in-plane-deformation, and could accordingly be assigned to the recorded signal at 738 cm^−1^ (Rastogi and Palafox, 2011).

Some significant variations in the SERS spectra are identified when compared with the normal Raman one as illustrated Figures 8B–D. For example, the N1−H stretching mode in the Au_6_-5FU_1 complex is strongly shifted to a lower energy region near 3188 cm^−1^, as compared to a corresponding value centered at 3548 cm^−1^ in free 5FU. The C=O stretching of carbonyl groups also changes significantly. Indeed, the peaks associated with the C2 = O and C4 = O bonds are significantly shifted, in opposite directions, from 1719 to 1675 cm^−1^ in free 5FU to 1620 (decreased by 99 cm^−1^) and 1697 cm^−1^ (increased by 22 cm^−1^) in Au_6_-5FU_1. Other vibrations including the N1−H bending *β*(N1−H), C6−H bending *β*(C6−H) and pyrimidine ring in-plane-deformation *δ*(ring), that are observed at 1399, 1318 and 737 cm^−1^ in Au_6_-5FU_1. However, they are slightly changed following adsorption on gold surfaces as they are not directly involved in the interactions (Table 3). More importantly, significant modifications of the intensities are also observed. In the SERS spectra, the N−H stretching modes turn out to be the highest intensity bands, rather than the overlapped bands around 800 cm^−1^ arising from the in-plane-deformation of pyrimidine ring as in free 5FU. Such vibrations that are now centered at 3200–3400 cm^−1^, represent the most significant increase. Gold nanoparticles synthetized using polyethylene glycol surfactants have been used to detect several drugs such as rhodamine, 5FU and doxorubicin. It has been shown that both *ν*(C=O) and *β*(N1−H) signals are significantly enhanced when coated on the gold substrate (Rastogi and Palafox, 2011).

### The drug release in target cell

**FIGURE 9:**
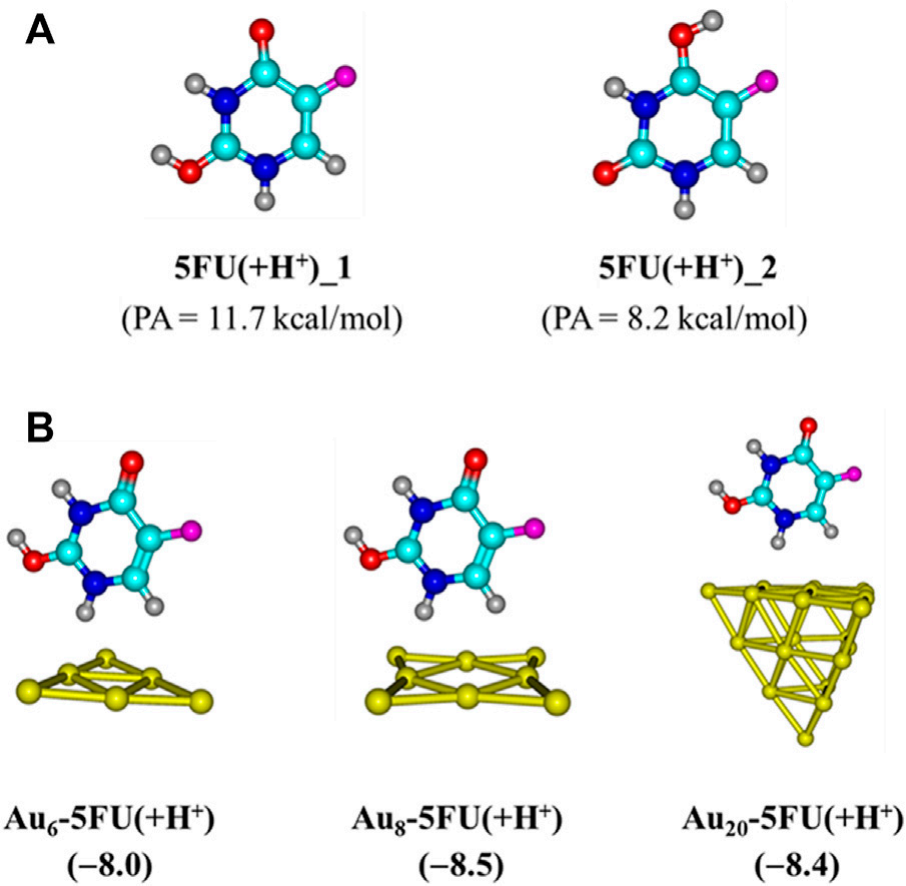
Optimal structures of 5FU in an acidic environment **(A)** and their complexes with gold clusters **(B)**. Values given in parentheses are binding energies (kcal/mol, PBE-D3/cc-pVTZ/pVDZ-PP + ZPE).

The drug releasing from the carrier in the target cells is one of the most important steps of a drug delivery process. In a human body, the drug can be separated from the carrier by either external or internal inducements such as the *p*H change or amino acid residues such as glutathione and cysteine (Rastogi et al., 2000). As a result of an extensive lactic production, tumor cells are typically more acidic than the normal ones. Indeed, the *p*H of cancer cells is normally smaller than 6 as compared to values around 7.4 in blood (Swietach et al., 2014). In an acidic media, protons can attack any rich-electron site of the 5FU molecule, but it appears that the C2 = O bond emerges as the most preferred site for protonation (Figure 9A). Therefore, we now examine the effects of protons on the stability of Au_N_-5FU products by protonation of the C2 = O, and then carry out geometry optimizations for the resulting Au_N_-5FU(+H^+^) complexes. As shown in Figure 9B, interactions between the 5FU molecule and gold atoms are now mainly predominated by weaker H-bonds, with a binding energy of around −8.0 kcal/mol as compared to corresponding values from −11 to −19 kcal/mol in an aqueous environment. It is expected that the 5FU drug binding to the carrier turns out to be much more breakable in an acidic environment, and thereby it is able to be released faster.

Another factor contributing crucially to drug release is an internal force related to amino acids present in the protein matrices (Ghosh et al., 2008). Some sulfur based-amino acid constituents such as cysteine and methionine are found to be the most preferred binding sites for metal particles (Ghosh et al., 2008; Swietach et al., 2014). Cysteine is a relatively strong acid with the acid dissociation constants of p*K*_1_ = 1.7 and p*K*_2_ = 8.3 (O'Neil, 2013). Therefore, in biological systems, it likely emerges in its deprotonated forms by a proton cleavage of either the carboxyl or thiol group (Eckhardt et al., 2013). For further insights into the drug release mechanism from the gold surfaces, we consider the following ligand exchange reaction:

$${Au}_{N}-5{FU}_{\left(aq\right)}+Cys{(-H^{+}{)}_{\left(aq\right)}\;\to \;{Au}_{N}-Cys(-H^{+}}_{\left(aq\right)}+5{FU}_{\left(aq\right)}$$

**FIGURE 10:**
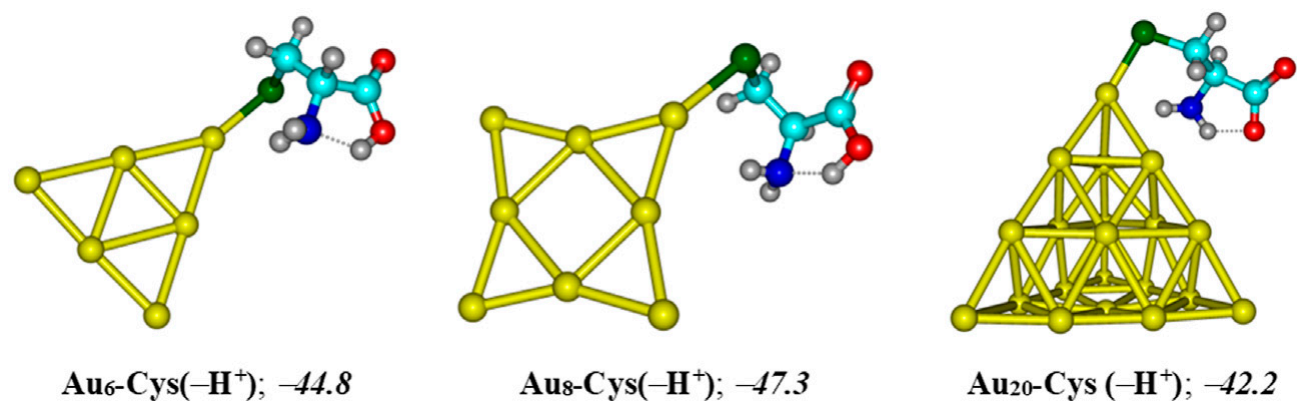
Resulting complexes of deprotonated cysteine Cys (−H^+^) with gold clusters Au_*N*_ (*N* = 6, 8, 20). Binding energies (kcal/mol) are computed at the PBE-D3/cc-pVTZ/cc-pVDZ-PP + ZPE level.

The resulting complexes of Au_N_ clusters with the deprotonated form of cysteine, denoted as Cys(−H^+^), are illustrated in Figure 10. In agreement with recent reports (Nhat et al., 2020a; Si et al., 2021; Le Guével et al., 2011), these systems are mainly stabilized due to a covalent bond between the S-atom of the thiol group and the corner Au atoms. In aqueous solution, the binding energies with Cys (−H^+^) are predicted to be around −45, −47, and −42 kcal/mol for Au_6_, Au_8_ and Au_20_, respectively, that are much more negative as compared to the corresponding values obtained for a 5FU adsorption. Under neutral condition, the largest binding energies of 5FU to Au_N_ cluster are situated in the range of −19 to −11 kcal/mol (Table 1), but significantly reduced to −8 kcal/mol in an acidic solution (Figure 9). The results clearly indicate that the gold affinity of cysteine residues is much stronger than that of the 5FU molecule. As a result, a drug release from a gold surface is expected to be spontaneous within the cancer cells upon protonation due to this internal inducement.

## Concluding remarks

In this theoretical study we attempted to elucidate the adsorption behaviours of the 5FU cancer drug on the Au nanostructured surfaces using three small gold clusters Au_*N*_ (*N* = 6, 8, 20) as model reactants. The structures, energetics and spectoscopic properties were analyzed thoroughly by employing a functional with dispersion correction, namely the PBE-B3. The effects of solvent in an aqueous solutiuon were also evaluated using the IEF-PCM model.

In both vacuum and aqueous solution, the 5FU molecule tends to bind with gold atoms via the oxygen atom of the C2 = O group. The resulting complexes are also partially stabilized by some weak N−H∙∙∙Au couplings. Nonetheless, in an acidic environment, the Au⋅⋅⋅H−X with X = O, C interaction turns out to be predominant, instead of the stronger gas phase covalent Au−O bond. Indeed, the largest binding energy is greatly declined from −24 kcal/mol in gas phase to −19 and −8 kcal/mol in neutral and acidic media, respectively. Following 5FU adsorption, the band gap of gold clusters is greatly modified giving rise to a significant change in the electronic properties. Analysis of the frontier MOs showed that a charge transfer from the 5FU molecule to the gold clusters constitutes the main ingredient of the 5FU−Au interaction. A mechanism for drug release from the gold surface has also been proposed and elucidated. Accordingly, such a process is triggered by either the cysteine residues present in protein matrices or the low *p*H in cancerous tissues.

An examination on the SERS spectra also allowed us to verify the preferable orientation of 5FU molecules on the gold surface. As being directly oriented to the gold surface, the N−H stretching mode turns out to be the most dominant factor responsible for the SERS enhancement of the drug molecules. These results show that it is possible to use SERS spectroscopy to detect the 5FU molecule in an aqueous medium through the use of gold nanoparticles.

## Data Availability

The original contributions presented in the study are included in the article/Supplementary Material, further inquiries can be directed to the corresponding author.
