# Supplementary material for: Binding mechanism and SERS spectra of 5-fluorouracil on gold clusters
Source: Front Chem. 2022 Dec 5;10:1050423. doi: 10.3389/fchem.2022.1050423 (PMC9760957; doi:10.3389/fchem.2022.1050423)
Supplement: Supplementary file 1 [file DataSheet1.docx]

**Supplementary Information**

**Binding mechanism and SERS spectra of 5-fluorouracil on gold clusters**

**Nguyen Thanh Si,^1^ Pham Vu Nhat^1^ and Minh Tho Nguyen^2,3,*^**

^1^Department of Chemistry, Can Tho University, Vietnam

^2^Laboratory for Chemical Computation and Modeling, Institute for Computational Science and Artificial Intelligence, Van Lang University, Ho Chi Minh City, Vietnam

^3^Faculty of Applied Technology, Van Lang University, Ho Chi Minh City, Vietnam

^*^ Email: [minhtho.nguyen@vlu.edu.vn](mailto:minhtho.nguyen@vlu.edu.vn)

**Figure S1.** Graphic representation of bond critical points in the most stables complexes Au*_N_*-5FU (*N* = 6, 8, 20)

**
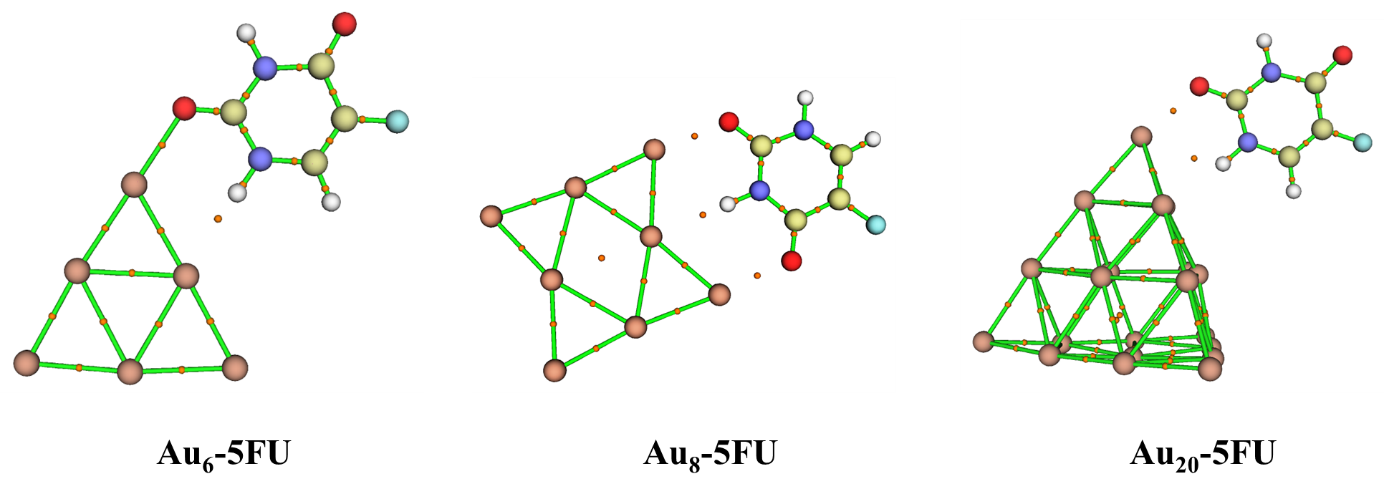
**

**Figure S2.** 2D reduced density gradient plots (isovalue 0.05 a.u.) of the most stables complexes Au*_N_*-5FU (*N* = 6, 8, 20).

The reduced density gradient is also a most efficient method for analyzing NCI. It enables to indentify and visualize zones of weak interactions, i.e. the van der Waals, hydrogen bonds and steric effect, in a molecular system. Blue zone indicates the presence of strong attractive interactions (ρ > 0 and λ_2_ < 0) such as hydrogen bond; red zone indicated the presence of strong repulsive interactions (ρ > 0 and λ_2_ > 0) such as steric effect and green zone shows the presence of weak interactions (ρ ≈ 0 and λ_2_ ≈ 0), i.e. van der Waals.

| 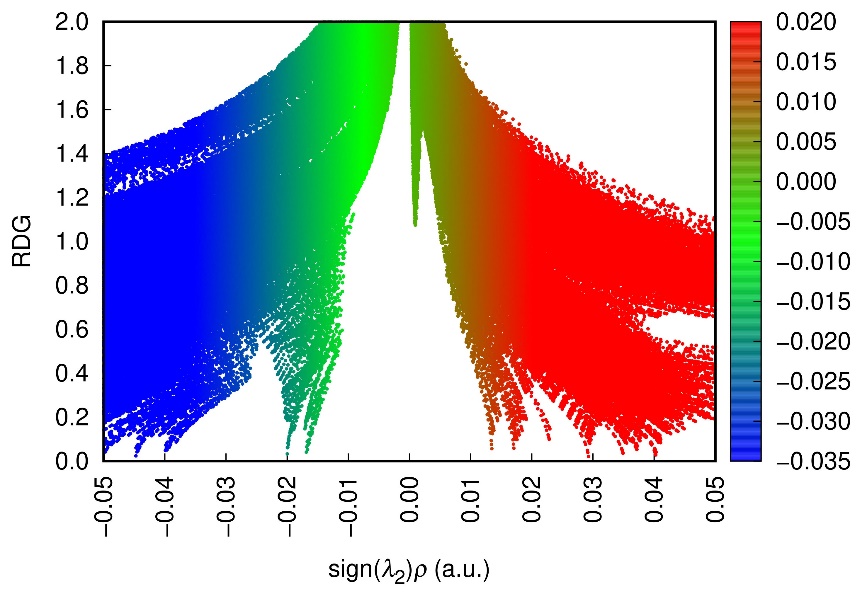  **Au_6_-5FU** |
| --- |
| 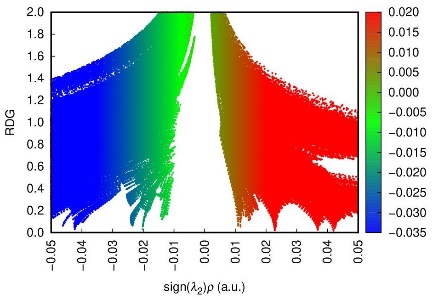  **Au_8_-5FU** |
| 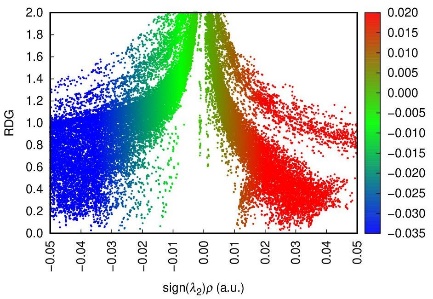  **Au_20_-5FU** |
| 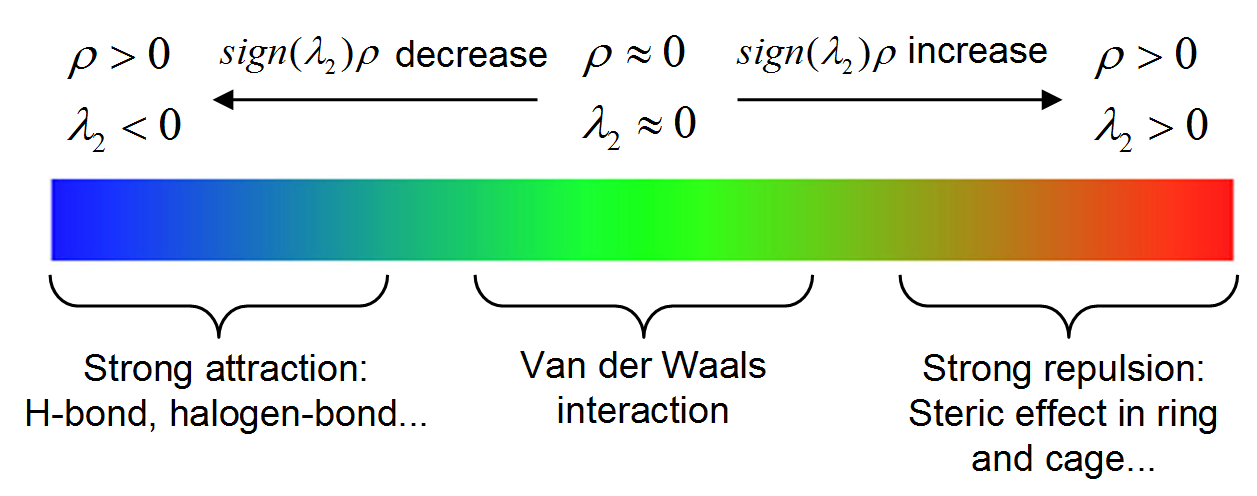 |

**Figure S3.** Born-Oppenheimer molecular dynamics simulations for **Au_6_-5FU** and **Au_8_-5FU** systems at 300 K. The relative nuclear kinetic energy and potential energy for Au_6_-5FU (a, b) and Au_8_-5FU (c, d) complexes

Simulation results displayed below show the connectivity between atoms in these systems remains unchanged during the simulation time, indicating that they are dynamically stable.

**
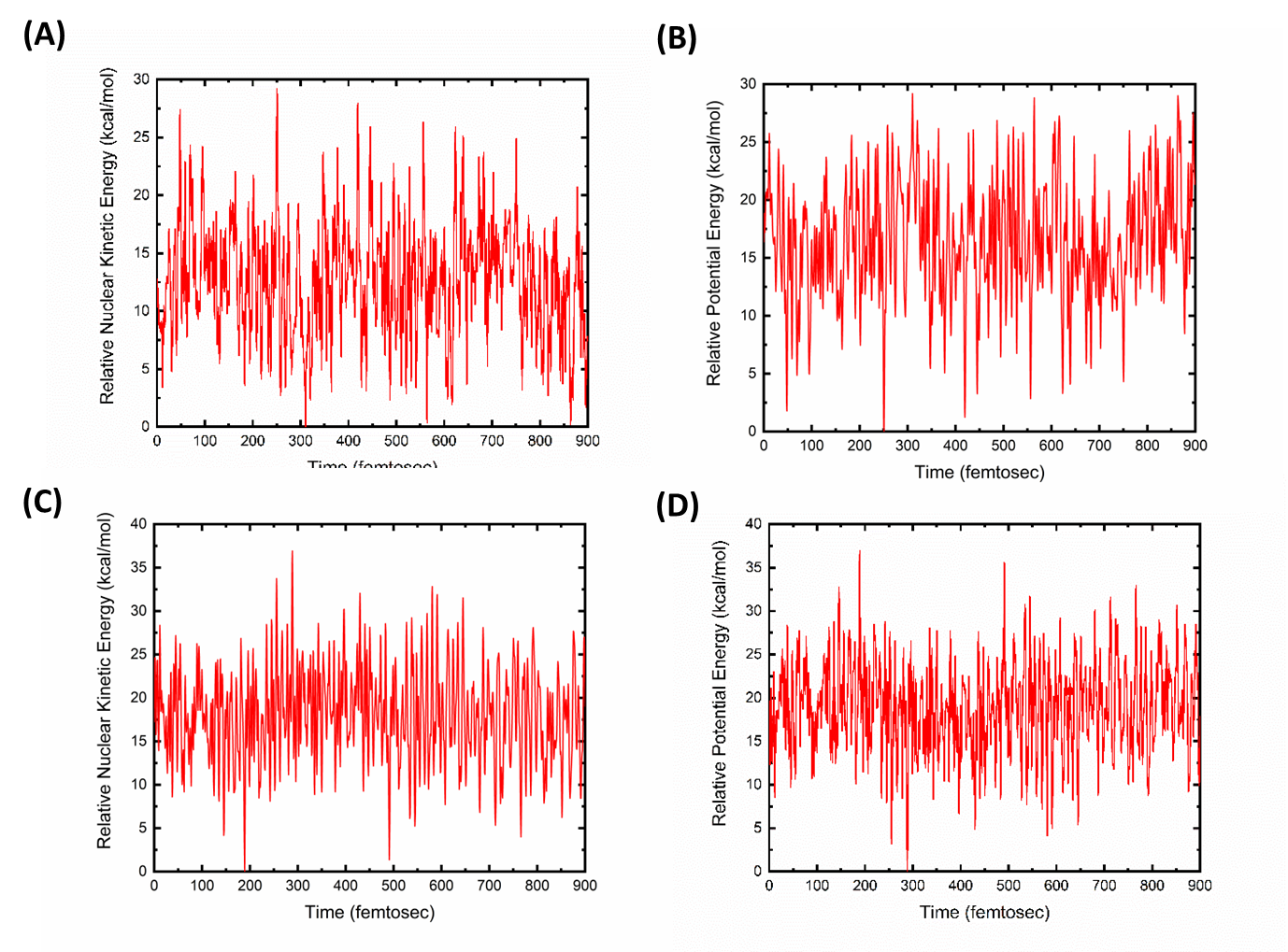
**

**Table S1.** The topological parameters of critical points including the electron density (*ρ*_r_, in hartree), Laplacian of electron density ($\nabla$^2^*ρ*_r_, in hartree), the density of potential energy (*V*_r_, in hartree), the density of kinetic energy (*G*_r_, in hartree), the density of total energy (*H*_r_, in hartree) and energy interaction (E_int_, kcal/mol) at the BCPs for the most stables complexes Au***_N_***-5FU (*N* = 6, 8, 20)

| Complexes | Bonds | *ρ*_r_ | $\nabla$^2^*ρ*_r_ | *G*_r_ | *V*_r_ | *H*_r_ | E_int_ |
| --- | --- | --- | --- | --- | --- | --- | --- |
| Au_6_-5FU | Au⋅⋅⋅O | 0.059 | 0.252 | 0.068 | -0.073 | -0.005 | -12.357 |
|  | Au⋅⋅⋅H | 0.020 | 0.037 | 0.011 | -0.012 | -0.002 | -3.716 |
| Au_8_-5FU | Au⋅⋅⋅O | 0.046 | 0.200 | 0.051 | -0.053 | -0.001 | -9.548 |
|  | Au⋅⋅⋅O | 0.053 | 0.230 | 0.061 | -0.064 | -0.003 | -11.064 |
|  | Au⋅⋅⋅H | 0.020 | 0.038 | 0.011 | -0.012 | -0.001 | -3.767 |
| Au_20_-5FU | Au⋅⋅⋅O | 0.049 | 0.205 | 0.053 | -0.056 | -0.002 | -10.221 |
|  | Au⋅⋅⋅H | 0.014 | 0.036 | 0.008 | -0.008 | 0.001 | -2.470 |

**Table S2.** The results of Energy Decomposition Analysis (EDA) between 5-fluorouracil (5FU) in gold clusters.

| Structures | ΔE_int_ | ΔE_orb_ | ΔE_steric_ |
| --- | --- | --- | --- |
| Au_6_-5FU | -16.15 | -30.62 | 14.46 |
| Au_8_-5FU | -24.32 | -41.05 | 16.73 |
| Au_20_-5FU | -14.55 | -27.29 | 12.74 |

*All in kcal/mol.

**Table S3**. Cartesian coordinates (in Å) of structures located from

PBE-D3/cc-pVDZ-PP/cc-pVTZ geometry optimizations.

| **Strucutres** | **Cartesian coordinates** |
| --- | --- |
| 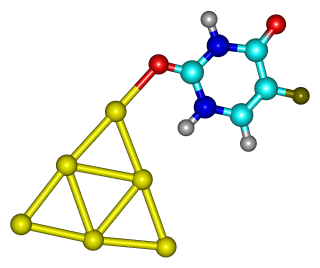  Au_6_-5FU_1 | 79 -0.329412000 3.032336000 -0.002367000  79 0.662050000 0.583102000 -0.001761000  79 -2.116074000 1.057686000 0.000487000  79 1.591737000 -1.916717000 -0.002010000  79 -3.633911000 -1.092254000 0.003418000  79 -1.013923000 -1.613948000 -0.000575000  9 7.112454000 2.162348000 0.012440000  6 6.309949000 1.091775000 0.008056000  6 4.963350000 1.225286000 0.010523000  6 6.950800000 -0.222136000 0.000807000  7 4.145588000 0.116403000 0.006240000  1 4.468778000 2.194605000 0.016091000  7 5.992130000 -1.270939000 -0.002854000  8 8.146546000 -0.453913000 -0.002165000  6 4.621105000 -1.168481000 -0.000549000  1 3.115901000 0.222468000 0.008225000  1 6.371686000 -2.216745000 -0.007684000  8 3.893374000 -2.184164000 -0.003276000 |
| 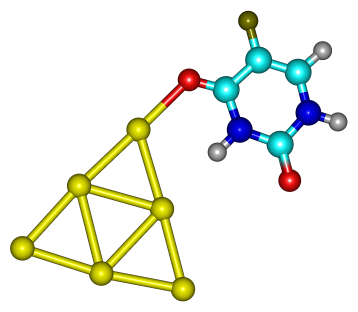  Au_6_-5FU_2 | 79 -1.793084000 -1.739016000 -0.000182000  79 0.829088000 -1.646241000 -0.000267000  79 -0.660479000 0.679493000 0.000118000  79 3.483501000 -1.349359000 -0.000448000  79 0.537482000 3.036742000 0.000562000  79 2.147529000 0.921845000 -0.000018000  8 -4.102308000 -1.863379000 -0.000101000  6 -4.796108000 -0.828085000 0.000108000  6 -6.246696000 -0.845335000 0.000125000  7 -4.249879000 0.444780000 0.000348000  6 -6.954394000 0.311173000 0.000326000  9 -6.859483000 -2.035794000 -0.000106000  6 -4.919996000 1.671364000 0.000629000  1 -3.215703000 0.500161000 0.000313000  7 -6.309666000 1.522028000 0.000544000  1 -8.042663000 0.319801000 0.000309000  8 -4.363941000 2.752835000 0.000708000  1 -6.835331000 2.390415000 0.000675000 |
| 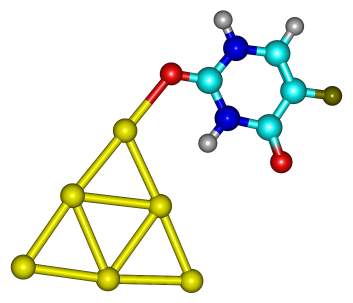  Au_6_-5FU_3 | 79 1.545561000 -2.023176000 -0.003075000  79 0.732001000 0.519547000 -0.002094000  79 -1.043667000 -1.602072000 -0.000319000  79 -0.160004000 3.007638000 -0.001469000  79 -3.636023000 -0.972352000 0.003331000  79 -2.022559000 1.112419000 0.000632000  8 3.860440000 -2.394217000 -0.000103000  6 4.638111000 -1.420491000 0.001655000  7 6.001934000 -1.616497000 0.004123000  7 4.251287000 -0.105307000 0.001337000  6 6.918638000 -0.583576000 0.006116000  1 6.307788000 -2.583987000 0.004414000  6 5.075413000 1.048240000 0.003105000  1 3.230118000 0.065281000 -0.000466000  6 6.499166000 0.701531000 0.005681000  1 7.972203000 -0.854203000 0.007996000  8 4.607843000 2.171981000 0.002471000  9 7.374856000 1.712320000 0.007568000 |
| 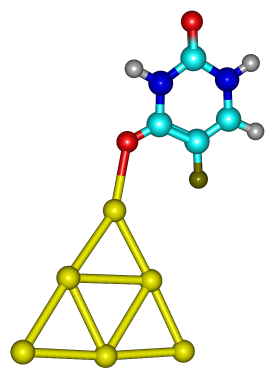  Au_6_-5FU_3 | 79 -2.069040000 -0.925526000 -0.193630000  79 0.489139000 -1.571594000 -0.041682000  79 -0.304149000 1.065044000 -0.083634000  79 3.102176000 -2.083956000 0.111281000  79 1.547628000 2.970563000 0.033322000  79 2.458911000 0.479951000 0.081653000  8 -4.373222000 -1.354799000 -0.240789000  6 -5.325883000 -0.580273000 -0.061723000  6 -5.288680000 0.870244000 -0.088753000  7 -6.595455000 -1.092916000 0.191506000  6 -6.411202000 1.600130000 0.126659000  9 -4.116804000 1.467813000 -0.336407000  6 -7.787749000 -0.398715000 0.419787000  1 -6.671416000 -2.108974000 0.220522000  7 -7.612451000 0.984591000 0.372261000  1 -6.397617000 2.688218000 0.109678000  8 -8.856738000 -0.937100000 0.636181000  1 -8.452166000 1.531506000 0.534610000 |
| 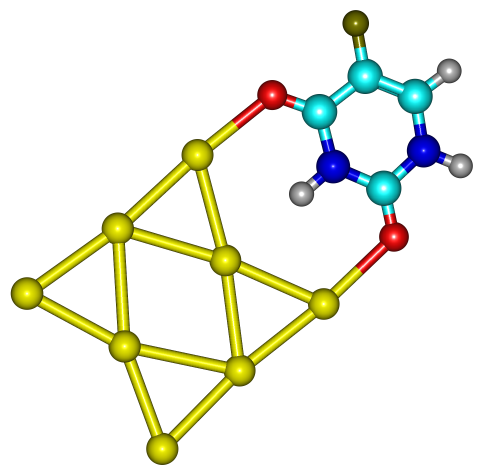  Au_8_-5FU_1 | 79 0.714955000 -2.268491000 0.000782000  79 -0.912542000 -0.090675000 0.005017000  79 0.402664000 2.285037000 0.000071000  79 2.267771000 0.124944000 -0.001052000  79 -2.221044000 2.231467000 0.002605000  79 3.355519000 -2.296780000 -0.000304000  79 -1.894098000 -2.568285000 -0.001083000  79 3.017709000 2.670008000 -0.002976000  8 -4.311296000 -2.615379000 -0.001813000  6 -4.969254000 -1.567733000 -0.003098000  7 -6.351849000 -1.600319000 -0.006489000  7 -4.433529000 -0.294381000 -0.001168000  6 -7.142747000 -0.475084000 -0.007683000  1 -6.765547000 -2.527707000 -0.008303000  6 -5.130799000 0.914089000 -0.001606000  1 -3.391664000 -0.235387000 0.001092000  6 -6.575941000 0.754784000 -0.005320000  1 -8.220888000 -0.619779000 -0.010470000  8 -4.570537000 2.019681000 0.000864000  9 -7.322321000 1.862702000 -0.006277000 |
| 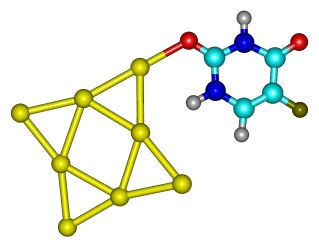  Au_8_-5FU_2 | 79 -0.396803000 -2.115201000 -0.073395000  79 1.074514000 0.175827000 -0.249575000  79 -0.861326000 2.105402000 -0.016065000  79 -2.450028000 -0.173186000 0.109961000  79 1.675000000 2.746325000 -0.524934000  79 -2.969717000 -2.749612000 -0.078799000  79 2.214734000 -2.216571000 -0.006968000  79 -3.449189000 2.267370000 0.441449000  8 4.479903000 -2.312010000 0.152006000  6 5.111554000 -1.238759000 0.285362000  7 4.533547000 0.001451000 0.296582000  7 6.476448000 -1.237333000 0.434283000  6 5.250965000 1.167264000 0.450420000  1 3.506466000 0.038158000 0.174784000  6 7.339606000 -0.119847000 0.598377000  1 6.930617000 -2.149642000 0.429798000  6 6.595598000 1.138279000 0.596588000  1 4.673091000 2.090490000 0.448358000  8 8.542905000 -0.260327000 0.721448000  9 7.301484000 2.264580000 0.747397000 |
| 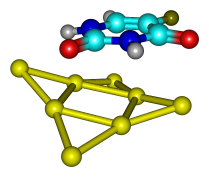  Au_8_-5FU_3 | 79 -1.838889000 -0.608039000 -0.483993000  79 -0.389532000 1.745863000 -0.578475000  79 2.031756000 0.432383000 -0.185705000  79 0.582383000 -1.930863000 -0.171383000  79 1.930614000 2.926267000 -1.033191000  79 -1.626687000 -3.220433000 -0.832095000  79 -2.971045000 1.725232000 -0.002851000  79 3.049970000 -1.809200000 0.787325000  6 0.660332000 1.298748000 2.940254000  7 0.429820000 -0.072600000 2.916006000  6 -0.821593000 -0.638044000 2.929368000  6 -1.928250000 0.133628000 3.046678000  6 -1.842528000 1.587840000 3.082085000  7 -0.511558000 2.040538000 3.073488000  1 -0.877041000 -1.719910000 2.826869000  1 1.239519000 -0.640958000 2.668627000  1 -0.388873000 3.051157000 3.050867000  8 1.773438000 1.796136000 2.849903000  8 -2.789980000 2.369091000 3.090376000  9 -3.154879000 -0.412920000 3.060403000 |
| 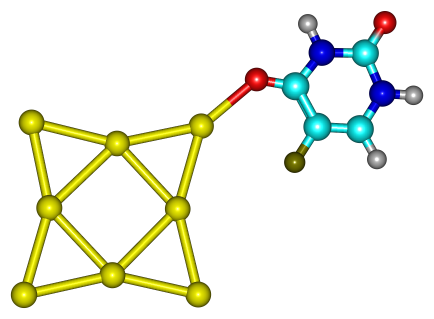  Au_8_-5FU_4 | 79 0.928449000 -0.945749000 0.000041000  79 0.291729000 1.701599000 -0.000044000  79 -2.407756000 0.983508000 -0.000034000  79 -1.742394000 -1.702290000 0.000055000  79 -1.643525000 3.512332000 -0.000133000  79 0.206529000 -3.494992000 0.000092000  79 2.807581000 0.916087000 -0.000015000  79 -4.264549000 -0.906150000 -0.000009000  8 5.052001000 1.348905000 -0.000042000  6 6.023365000 0.573420000 0.000008000  6 5.989951000 -0.876578000 0.000100000  7 7.312017000 1.095874000 -0.000011000  6 7.139254000 -1.597502000 0.000148000  9 4.798557000 -1.485241000 0.000130000  6 8.531776000 0.410830000 0.000046000  1 7.384435000 2.112723000 -0.000076000  7 8.360479000 -0.973754000 0.000118000  1 7.130003000 -2.685785000 0.000209000  8 9.617540000 0.958097000 0.000001000  1 9.219582000 -1.514864000 0.000153000 |
| 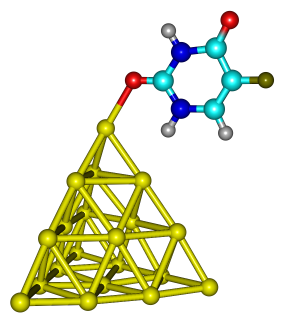  Au_20_-5FU_1 | 79 0.947105000 3.102084000 0.011239000  79 2.420477000 1.098258000 -1.492324000  79 1.209001000 -0.872439000 -2.813343000  79 -1.754453000 -1.009935000 -2.813317000  79 -3.189708000 0.807139000 -1.465764000  79 -1.750411000 3.005259000 0.012649000  79 -0.392700000 1.117159000 -1.551603000  79 2.423933000 1.086671000 1.497182000  79 -0.389300000 1.102946000 1.557459000  79 -3.186754000 0.796284000 1.475581000  79 1.452659000 -1.105065000 -0.005007000  79 -0.144108000 -3.024807000 -1.344870000  79 -1.850876000 -1.212689000 -0.003615000  79 -1.750073000 -1.031097000 2.807638000  79 1.214621000 -0.893717000 2.805913000  79 -0.142089000 -3.034130000 1.323046000  79 3.638806000 3.031628000 0.008443000  79 -0.181966000 -2.850373000 -4.063286000  79 -4.474246000 2.687436000 0.013205000  79 -0.176006000 -2.879918000 4.042885000  8 6.013049000 2.653075000 0.005311000  6 6.440468000 1.481283000 0.001511000  7 5.641300000 0.365415000 -0.004064000  7 7.788980000 1.207817000 0.002770000  6 6.129029000 -0.922557000 -0.008219000  1 4.626762000 0.526797000 -0.005474000  6 8.430232000 -0.059812000 -0.001348000  1 8.409661000 2.015945000 0.006764000  6 7.461743000 -1.154528000 -0.006949000  1 5.388579000 -1.720525000 -0.012432000  8 9.644688000 -0.156808000 -0.000115000  9 7.949264000 -2.400678000 -0.010896000 |
| 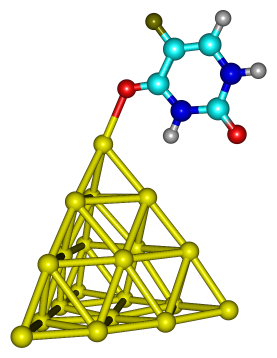  Au_20_-5FU_2 | 79 -2.951391000 0.357512000 -0.000332000  79 -1.915825000 -1.967660000 1.431276000  79 0.373286000 -1.763720000 2.818264000  79 1.899739000 0.800402000 2.817995000  79 0.928397000 2.905588000 1.481386000  79 -1.635199000 2.673549000 0.000140000  79 -0.644717000 0.566495000 1.619403000  79 -1.915510000 -1.967227000 -1.432295000  79 -0.644624000 0.567100000 -1.619877000  79 0.928697000 2.905981000 -1.480417000  79 0.526014000 -1.932893000 -0.000224000  79 2.966951000 -1.523186000 1.339887000  79 2.109944000 0.811809000 0.000322000  79 1.900331000 0.801167000 -2.817381000  79 0.373924000 -1.762892000 -2.818683000  79 2.967236000 -1.522816000 -1.339680000  79 -4.236301000 -2.055512000 -0.000802000  79 2.793407000 -1.451284000 4.052328000  79 -0.119745000 4.936770000 0.000657000  79 2.794340000 -1.450160000 -4.052132000  8 -6.591688000 -1.988317000 -0.000610000  6 -7.206761000 -0.904462000 -0.000177000  6 -8.654666000 -0.813809000 -0.000016000  7 -6.569387000 0.325592000 0.000175000  6 -9.275571000 0.391755000 0.000443000  9 -9.353269000 -1.955672000 -0.000341000  6 -7.147560000 1.599384000 0.000662000  1 -5.536214000 0.310711000 0.000059000  7 -8.544195000 1.551947000 0.000763000  1 -10.360323000 0.480065000 0.000567000  8 -6.512585000 2.635895000 0.000919000  1 -9.004801000 2.456637000 0.001098000 |
| 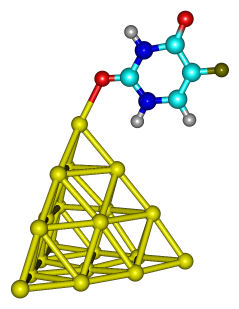  Au_20_-5FU_3 | 79 -1.729179000 -2.166418000 -1.413675000  79 -2.936344000 0.091770000 -0.019341000  79 -1.822795000 2.504441000 -0.050116000  79 0.708840000 2.923540000 -1.526544000  79 1.863001000 0.887789000 -2.826222000  79 0.543903000 -1.793067000 -2.794801000  79 -0.679291000 0.469702000 -1.675392000  79 -1.744154000 -2.122325000 1.457268000  79 0.684179000 -1.862469000 0.030457000  79 3.101195000 -1.319150000 -1.305480000  79 -0.697867000 0.520856000 1.649170000  79 0.692503000 2.969132000 1.439956000  79 2.029437000 0.948154000 -0.005291000  79 3.086586000 -1.277890000 1.375541000  79 0.513520000 -1.706222000 2.850863000  79 1.831851000 0.974471000 2.814160000  79 -4.042635000 -2.402259000 0.012876000  79 -0.507089000 4.885968000 -0.079808000  79 2.938628000 -1.304250000 -4.019588000  79 2.894590000 -1.179460000 4.086030000  8 -6.402092000 -2.373111000 0.014747000  6 -7.011356000 -1.282472000 0.007490000  7 -6.398971000 -0.056688000 -0.003002000  7 -8.385147000 -1.231845000 0.009601000  6 -7.088162000 1.136591000 -0.011001000  1 -5.365147000 -0.056395000 -0.005333000  6 -9.222199000 -0.083769000 0.002165000  1 -8.866564000 -2.130092000 0.017458000  6 -8.440903000 1.151894000 -0.008631000  1 -6.487166000 2.044006000 -0.019119000  8 -10.436009000 -0.182473000 0.005391000  9 -9.121481000 2.303394000 -0.016179000 |
| 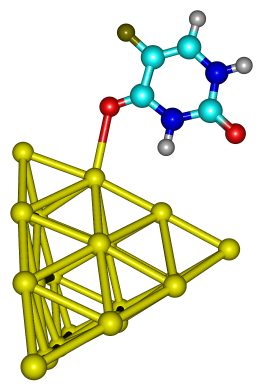  Au_20_-5FU_4 | 79 -0.727087000 -2.797161000 1.468509000  79 -0.726808000 -2.797406000 -1.468348000  79 1.199942000 -1.520331000 -2.813067000  79 3.339278000 -0.074405000 -1.336659000  79 3.339035000 -0.074232000 1.337186000  79 1.199439000 -1.519938000 2.813401000  79 1.411113000 -1.653065000 0.000194000  79 -2.850960000 -1.226139000 -0.000192000  79 -0.818649000 0.011984000 1.534209000  79 1.255901000 1.432761000 2.818211000  79 -0.818263000 0.011742000 -1.534224000  79 1.256386000 1.432376000 -2.818241000  79 1.463428000 1.563227000 -0.000007000  79 -0.566514000 2.860882000 1.484354000  79 -2.678171000 1.459683000 -0.000359000  79 -0.566263000 2.860675000 -1.484898000  79 -2.682938000 -3.968585000 -0.000012000  79 3.158049000 -0.074944000 -4.051566000  79 3.157341000 -0.074408000 4.052062000  79 -2.455868000 4.157293000 -0.000521000  8 -5.442296000 -1.779969000 -0.000076000  6 -6.278846000 -0.870135000 -0.000055000  6 -7.721314000 -1.074802000 0.000060000  7 -5.920836000 0.475133000 -0.000138000  6 -8.576932000 -0.024724000 0.000068000  9 -8.171895000 -2.337077000 0.000136000  6 -6.745142000 1.596561000 -0.000082000  1 -4.902205000 0.671238000 -0.000223000  7 -8.101372000 1.265208000 -0.000016000  1 -9.656713000 -0.160710000 0.000135000  8 -6.338765000 2.746812000 -0.000231000  1 -8.739619000 2.053983000 -0.000031000 |
